# Two-Year Outcomes for the Active and Healthy Families Pediatric Obesity Group Intervention for Families in an Emerging Latinx Community: a Mixed Methods Study

**DOI:** 10.1007/s40615-025-02420-x

**Published:** 2025-04-23

**Authors:** Jaime La Charite, Lisa Ross DeCamp, Laura Prichett, Amanda Grace Finney, Jenny Y. Chen, Albert E. Holler, Yoon Ji Moon, Alexa Mullins, Rafael Ospino, Kori Porosnicu Rodriguez, Sarah Polk

**Affiliations:** 1 The Johns Hopkins University School of Medicine, 601 N Caroline St, Baltimore, MD USA; 2 Department of General Internal Medicine, University of California, Los Angeles, 1100 Glendon Ave. Suite 900, Los Angeles, CA 90024 USA; 3 Children’s Hospital Colorado, Aurora, CO USA; 4 Department of Pediatrics, University of Colorado School of Medicine, Aurora, CO USA; 5 Adult and Child Center for Outcomes Research and Delivery Science, Aurora, CO USA; 6 Department of Pediatrics, Johns Hopkins University School of Medicine, Baltimore, MD USA; 7 Department of General Pediatrics, Children’s Hospital of Philadelphia, 3401 Civic Center Boulevard, Philadelphia, PA 19104 USA; 8 Department of Pediatrics, Children’s Hospital of Philadelphia, Philadelphia, PA USA; 9 Department of Neurology, Johns Hopkins University School of Medicine, 600 North Wolfe Street, Baltimore, MD USA; 10 Centro SOL, Johns Hopkins University, 5200 Eastern Ave, Baltimore, MD 21224 USA; 11 Center for Salud, Health and Opportunity for Latine, Baltimore, USA

**Keywords:** Pediatric obesity, Latinx, Immigrant health, Active and Healthy Families, Mixed method study

## Abstract

**Introduction:**

Childhood obesity and its comorbidities disproportionately affect Latinos, but there are not clear interventions to narrow the disparity and have a lasting impact. Our study aims to assess the 2-year outcomes and explore the potential mechanisms of behavior change, along with the barriers to sustaining those behaviors, among participants of a family-based, culturally tailored Spanish-language weight management program.

**Methods:**

We conducted a mixed methods study comprised of a retrospective secondary data analysis and semi-structured interviews. The intervention, Active and Healthy Families, consisted of eight biweekly group sessions for child-caregiver dyads in an emerging Latinx community. We extracted clinic visit data from child participants and matched controls from 2017 to 2021. We compared normalized BMI measurements (BMIpct95) between the intervention and control arm participants using mixed effects linear regression modeling from the start to 2 years post-intervention. We conducted caregiver interviews from 2020 to 2021 after intervention participation and performed a thematic analysis.

**Results:**

Intervention participation for the 40 AHF child participants was associated with a lower-than-expected average BMIpct95 compared to controls at two years post-intervention. Interview themes included as follows: (1) caregivers felt responsible for their children’s health; (2) families acquired new knowledge that they applied; (3) the group format facilitated mutual support and sharing; and (4) the COVID-19 pandemic exposed barriers to maintaining behavior change.

**Conclusion:**

The AHF intervention may effectively support long-term pediatric weight loss in an emerging Latinx community. Parents offered insights into key intervention components that may facilitate behavior change and identified opportunities to reduce barriers to sustain those behaviors.

## Introduction

Latinx children have the highest prevalence of being overweight and obese (26.2%) compared to non-Hispanic Black (24.8%), White (16.6%), and Asian (9.0%) children [1]. The rate of obesity also increased more rapidly among Latinx youth than White youth during the pandemic, especially among children from lower-income households [2]. Factors believed to be driving these disparities include differences in household income/food security, environmental conditions, parental education, parent health behaviors (e.g., parent feeding practices, modeling), quality of meals, level of physical activity, screen time, and sleep duration [3, 4]. Effective interventions are urgently needed to counter disparities in childhood obesity.

There is no consensus on which interventions are needed to narrow the gap in weight trends between Latinx and non-Latinx children. A recent meta-analysis of pediatric obesity prevention and treatment interventions among Latinx youth revealed mixed and minimal effects and limited implementation [5]. Intervention effectiveness varied based on the type of intervention (diet-focused interventions were more effective than physical activity-focused ones), age (more effective during infancy and adolescence), and child characteristics (more effective for higher-income youth) [5]. These interventions had less impact on physical activity, fruit, vegetable, sugar-sweetened beverage intake, and sedentary behavior [5]. Dissemination may have been hampered by poor intervention packaging reducing ease of uptake [5]. Moreover, only nine interventions were family-centered and culturally tailored, even though parental involvement has been seen as a critical component of improving a Latinx child’s weight status [6]. Furthermore, most of the available robust evidence is for localities with densely populated Latinx communities, while gaps remain regarding the efficacy of similar programs in states with smaller but growing Latinx populations without extensive social and support structures in place [6]. The long-term impacts of these interventions, especially in the face of the social isolation policies and emotional stress of the COVID- 19 pandemic, are also not well documented [6, 7].

Long-term evaluation of family-based, culturally tailored programs in emerging—or nascent—Latinx communities may help inform strategies to meet the American Academy of Pediatrics (AAP) and the United States Preventative Services Taskforce (USPSTF) guidelines that children and adolescents with obesity should be referred to comprehensive, intensive health behavior and lifestyle treatment [8, 9]. One such program is Active and Healthy Families (AHF), a behavioral-theory-based, culturally tailored, Spanish-language weight management program for overweight and obese Latinx children and their caregivers [10]. The intervention has demonstrated short-term in reducing child body mass index (BMI) and triglycerides for children in low-income immigrant Latinx families in Contra Costa, CA [10]. The program’s long-term outcomes, feasibility, and acceptability in other Latinx communities have not been tested. Understanding whether it is possible to disseminate and scale AHF while maintaining its effectiveness is a critical next step for incorporating it as a program option that fits the AAP and USPSTF recommendations. This is particularly relevant for emerging immigrant communities, defined as areas with a significant population increase in a non-Anglo-Celtic immigrant group, which may be more vulnerable due to a lack of family networks and supports compared to established communities [11].

We conducted a mixed methods study with two aims. For the quantitative component, our first aim was to test the degree to which AHF participation alters the 2-year BMI trajectory of 4 to 14-year-old children meeting criteria identifying them as overweight or obese in an emerging Latinx immigrant community. We hypothesized that pediatric participants enrolled in AHF would demonstrate a lower-than-expected BMI trend (primary outcome) at two years post-intervention compared to matched controls. For the qualitative component, our second aim was to explore potential mechanisms through which AHF might influence behavior change, as well as to identify ongoing barriers to maintaining behavior change by interviewing parent intervention participants about their experiences during and after intervention participation.

## Methods

### Study Design

**Fig. 1:**
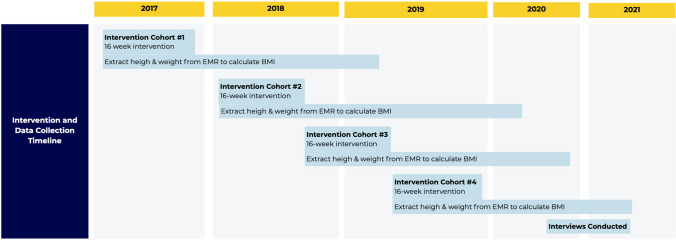
Intervention and data collection timeline. EMR = electronic medical record; BMI = body mass index

For this study, four cohorts of parent–child dyads (40 parent–child dyads in total) participated in the AHF intervention in Baltimore, MD, from 2017 to 2019. From 2020 to 2022, we evaluated the AHF intervention using a mixed methods approach. For the quantitative aim, we conducted a retrospective cohort study of intervention and matched control participants using secondary electronic medical record (EMR) height and weight data from 2017 to 2021. This study portion was used to assess the impact of the intervention on the child’s 2-year BMI trend. For the qualitative aim, we conducted semi-structured interviews with parents who participated in at least one of the AHF intervention sessions from 2020 to 2021. Interviews were grounded in descriptive phenomenological methodology to describe what our research team perceived as parent participants shared what and how they experienced the intervention and post-intervention period in an open-ended manner not based on assumptions [12].We chose this approach since it can be a useful technique to learn from the experience of others [13]. Once the interviews were transcribed, we used thematic analysis to “identify, analyze, and report patterns (themes) within the data” using a standard set of steps described below [14]. This study portion offered insights into the potential facilitators and barriers that may have influenced the BMI trend for the intervention arm (see Fig. 1 for the intervention and data collection timeline).

When feasible, the TREND guidelines for reporting nonrandomized/quasi-experimental study designs were followed when writing this manuscript. The study was approved by the institutional review board of the Johns Hopkins School of Medicine (IRB00136080).

### Intervention and Setting

This study’s intervention replicated the original AHF intervention [10, 15], which consisted of eight biweekly 2-h group sessions for parent–child dyads over 16 weeks. The content for the sessions was based on evidence-based practice guidelines. Sessions followed a curriculum and set of objectives, providing as follows: (1) practical nutrition and physical activity information; (2) problem-solving skills to overcome barriers and manage stressors; and (3) positive parenting skills to promote behavior change. Each session offered hands-on activities and children’s breakout groups. Participants received take-home materials to facilitate behavior change. Sessions were co-facilitated by university contracted staff, including a pediatrician, registered nurse, and promotora (community health worker). Funding for staff and programming was primarily grant-supported. We delivered the intervention to four cohorts of about 10 parent–child dyads each from September 2017 until September 2019.

For this study, the intervention took place at a hospital-based pediatric primary care clinic in southeast Baltimore, MD. Baltimore is a nontraditional, but emerging immigrant city for Latinx populations, with immigrants primarily arriving from Mexico and Central America. This setting differs from where AHF was originally tested in California, a state with a long history of immigrant community formation. The Baltimore clinic serves predominantly Latinx children in low-income households whose parents have a non-English language preference.

### Intervention Participants

Pediatric clinicians in the aforementioned Baltimore clinic could refer pediatric patients to the intervention through their electronic medical record based on the following criteria: children had to be (1) aged 5–12 years, (2) have a BMI at the 85 th percentile or greater, and (3) a caregiver self-identifying as Latinx or Hispanic and speaking Spanish. Seven children just beyond the age cut-offs (aged 4 and 13–14 years) were included in the intervention at the request of their pediatric clinician. We elected to include these children outside the age ranges to facilitate participation by families with siblings of varying ages and to avoid turning families away who may still benefit from the intervention. Exclusion criteria included a parent or child medical contraindication to diet/physical activity modification. Records were not obtained to identify the number and characteristics of all the children referred to the intervention. A total of 40 parent–child dyads participated in the intervention.

### Aim 1: Quantitative Evaluation of AHF Intervention on Child’s 2-Year BMI Trajectory

#### Data Source

In 2022, we extracted demographic (age, sex, preferred healthcare language) and medical (clinic encounter height and weight) clinic EMR data from 2017 to 2021 for the study participants in the intervention and control arms.

#### Study Participants

For the intervention arm, the study inclusion criteria were any child aged 4–14 years who participated in at least one session of the Baltimore AHF intervention from 2017 to 2019. As a pilot study, power calculations were not performed, and intervention enrollment stopped in 2019 because of the COVID- 19 pandemic.

For the control arm, we identified participants from the same clinic that the intervention participants attended. We extracted data from the clinic’s EMR for five control participants to one intervention participant using nearest neighbor matching with replacement according to the following matching criteria: child age, child sex, the child’s BMI at the date of the baseline encounter, and Spanish as the preferred healthcare language. They also needed at least three clinical encounters with height and weight data from 2017 to 2021. The baseline encounter was defined as the clinical encounter for the child in the control group that occurred closest in date to the starting intervention date for the matched child in the intervention group. Following this matching process, 153 unique participants were identified for the control arm.

#### Measures

##### Primary Outcome

Our outcome measure was BMIpct95, a measure normalized to the 95 th percentile of BMI (e.g., BMI equal to the 95 th percentile would have a BMIpct95 of 100) (1) and a valid way to assess intervention effectiveness [16]. We used the EMR-extracted height and weight data to calculate 2-year trends of the BMIpct95 for each intervention and control participant using the Center for Disease Control and Prevention’s SAS statistical software code [17]. We calculated the baseline BMIpct95 based on clinical encounter data that occurred closest to the intervention start, whether before or after the intervention start date. Missing height and weight values were filled in using a longitudinal simple imputation approach previously applied to BMI estimates [18]. This method estimates missing data points based on the participant’s available height or weight measurements over time, allowing for both interpolation (between known values) and extrapolation (beyond known values) as needed.

##### Primary Predictor

We defined our primary predictor variable as whether the child received the intervention versus usual care. Usual care was defined as an annual physical exam with a scheduled clinical encounter 6 months later to review lifestyle counseling.

##### Covariates

Age at baseline (in years), sex (male/female), and baseline BMIpct95.

#### Data Analysis

##### Descriptive Analyses

Characteristics of participants in the intervention and control groups were compared using Wilcoxon rank-sum tests for non-normal continuous variables and Fisher’s exact tests for normally distributed categorical variables.

##### Regression Analyses

To examine differences between BMIpct95 in the intervention versus control group, we used a series of mixed multilevel regression models, which allowed for random slopes as well as random intercepts. All BMIpct95 values from the closest visit to the start of the intervention (for the intervention participant) or the matched index encounter (for the matched control participant) up to 24 months after the intervention start or baseline encounter date were included in the mixed multilevel model. Models were adjusted for age, sex, and baseline BMIpct95. Variable selection for the model was guided by prior evidence and clinical insight, and model structure was guided by knowledge of disease and output from model comparisons using likelihood ratio tests. All analyses were performed using Stata 17.0 (StataCorp, College Station, TX).

##### Sensitivity Analyses

We performed three sensitivity analyses: (1) we narrowed the sample to children who met the original intervention age inclusion criteria of 5–12 years since the intervention was tailored to this age group; (2) we restricted the baseline encounter observations to those on or after the intervention start date (rather than allowing baseline encounter observations prior to the intervention start date); and (3) we dropped all BMIpct95 data after February 28, 2020, to minimize the effects of the COVID- 19 pandemic as a potential confounder.

### Aim 2: Qualitative Evaluation to Identify Mechanisms and Barriers for Behavior Change

#### Research Team and Reflexivity

Six bilingual (four female, two male) medical student research associates (RAs) with bachelor’s degrees conducted the interviews and performed the qualitative analysis. All RAs were trained in qualitative interviewing, thematic analysis, and coding in Dedoose. The first and senior author provided oversight and feedback throughout the process. Our team size allowed us to accommodate RA time constraints. The RAs did not have existing relationships with the study participants.

#### Participant Selection

From November 2020 to January 2021, the RAs called all 40 parent intervention participants to invite them for an interview to discuss their experience during and following their participation in one of the 2017–2019 intervention cohorts. We elected to conduct the interviews during this period since intervention enrollment had concluded, allowing us to interview parents 1 to 3 years after their participation and align this timing roughly with the quantitative data. We were concerned that waiting until after the quantitative results were finalized would make it difficult for parents to recall their time in the intervention. Parent participants were informed that the research was intended to understand their experiences during the intervention and its impact on their family, responses would remain confidential, and their participation decision would not impact their medical care. Fourteen parents consented and were enrolled in the interview portion of the study.

#### Data Collection and Setting

RAs scheduled a meeting with each enrolled parent using a virtual meeting platform. The interviewer was in a private room and participants were encouraged to do the same to protect their privacy. For the first 10 min, the RA administered a pre-interview survey consisting of demographic, medical, and health literacy questions. The RA then conducted a 30-min semi-structured interview with the parent using an interview guide developed by the study team. Participants were asked to describe their motivation to participate, overall experience in the AHF intervention, short- and long-term household changes in knowledge and behaviors related to nutrition and physical activity, and the COVID- 19 pandemic’s impact on their family’s health habits. Participants received a gift card for their time. Audio recordings of the interviews were transcribed verbatim in Spanish using a transcription company compliant with the Health Insurance Portability and Accountability Act (HIPAA).

#### Data Analysis

Analysis followed five steps: (1) data familiarization, (2) initial coding generation and reduction of codes, (3) searching for themes, (4) reviewing and refining themes, and (5) theme definition and labeling (2). The coders read through all the transcripts and took notes about prominent ideas. Coders developed a preliminary master codebook using discussion-based consensus [19]. Codes were refined in an iterative process as additional transcripts were coded and potential changes were presented to the team for discussion. Team members independently coded assigned transcripts and subsequently reconciled their codes with a second team member after they established consensus regarding discrepancies, resulting in a consensus-coded transcript. After coding was complete, team members individually searched for themes by reviewing the coded transcripts and code frequencies. These candidate themes were reduced to four main themes via team consensus. We used Dedoose qualitative data management and analysis software version 8.3.47 (SocioCultural Research Consultants, LLC; Los Angeles, CA).

## Results

### Aim 1: Quantitative Evaluation of AHF Intervention on Child’s 2-Year BMI Trajectory

#### Demographics (Full Results in Table 1)

**Table 1:** Intervention and control participant characteristics at baseline (initiation of intervention)

Variable	Control	Intervention	*p*-value^a^
*N*	153	40	
BMIpct95^a^ at baseline, median (IQR)	118 (109, 125)	125 (111, 135)	0.031*
Female, *N* (%)	77 (50%)	20 (50%)	1.00
Age category, *N* (%)			
Age 4–6 years	14 (9%)	3 (8%)	1.00
Age 7–10 years	81 (53%)	21 (53%)	
Age 11–14 years	58 (38%)	16 (40%)	

^*^p-value < 0.05

^a^Wilcoxon rank-sum test was used to compare medians and Fisher’s exact test was used to compare proportions

BMIpct95 is a measure normalized to the 95 th percentile of BMI (meaning that a patient with a BMI equal to the 95 th percentile would have a %BMIp95 of 100). The* p*-values were calculated based on bivariate analyses comparing the baseline characteristics between the intervention and control group participants

Forty children ages 4 to 14 years (mean age eight years) participated in the AHF intervention (ages 4–6 years: three children, ages 7–10 years: 21 children, and ages 11–14 years: 16 children). There was an even split among boys and girls. Differences in the child’s age and sex were not significant between the control and intervention groups. All caregivers identified their children as Latinx/Hispanic. No participants identified as multi-ethnic/racial. At the start of the intervention, the average BMIpct95 was 127% (SD 23). The average baseline BMIpct95 was significantly higher in the intervention group (127%) compared to the control group (118%) (*p* = 0.01). All participating caregivers identified themselves as their child’s mother.

#### Regression Analysis (Full Results in Table 2)

**Table 2:** Intention to treat analysis showing mixed multilevel regression model coefficients with their 95% confidence intervals comparing the BMIpct95 trend for the intervention and control participants adjusted for age at baseline, sex, and BMIpct95 at baseline

	Variable	*N*	Coefficient	95% confidence interval	*p*-value
Overall	Intervention		− 1.01	(− 1.89, − 0.13)	0.03*
	Months from intervention	193	0.12	(0.05, 0.20)	0.001***
	Intervention * months from intervention		− 0.18	(− 0.35, − 0.02)	0.03*
Females	Intervention		− 1.29	(− 2.49, − 0.09)	0.04*
	Months from intervention	97	0.11	(− 0.003, 0.21)	0.06
	Intervention * months from intervention		− 0.17	(− 0.40, 0.06)	0.14
Males	Intervention		− 0.62	(− 1.89, 0.64)	0.33
	Months from intervention	96	0.15	(0.04, 0.25)	0.01**
	Intervention * months from intervention		− 0.19	(− 0.43, 0.05)	0.12
Age 4–6 years	Intervention		− 1.17	(− 3.38, 1.03)	0.30
	Months from intervention	17	0.15	(− 0.07, 0.37)	0.18
	Intervention * months from intervention		0.01	(− 0.47, 0.49)	0.97
Age 7–10 years	Intervention		− 1.04	(− 2.26, 0.18)	0.09
	Months from intervention	102	0.11	(0.01, 0.22)	0.04
	Intervention * months from intervention		− 0.17	(− 0.40, 0.06)	0.15
Age 11–14 years	Intervention		− 0.69	(− 2.15, 0.76)	0.35
	Months from intervention	74	0.14	(0.02, 0.26)	0.03*
	Intervention * months from intervention		− 0.23	(− 0.51, 0.04)	0.09

^*^p-value ≤ 0.05, ** ≤ 0.01, *** ≤ 0.001

BMIpct95 is a measure normalized to the 95 th percentile of BMI (meaning that a patient with a BMI equal to the 95 th percentile would have a %BMIp95 of 100)

**Fig. 2:**
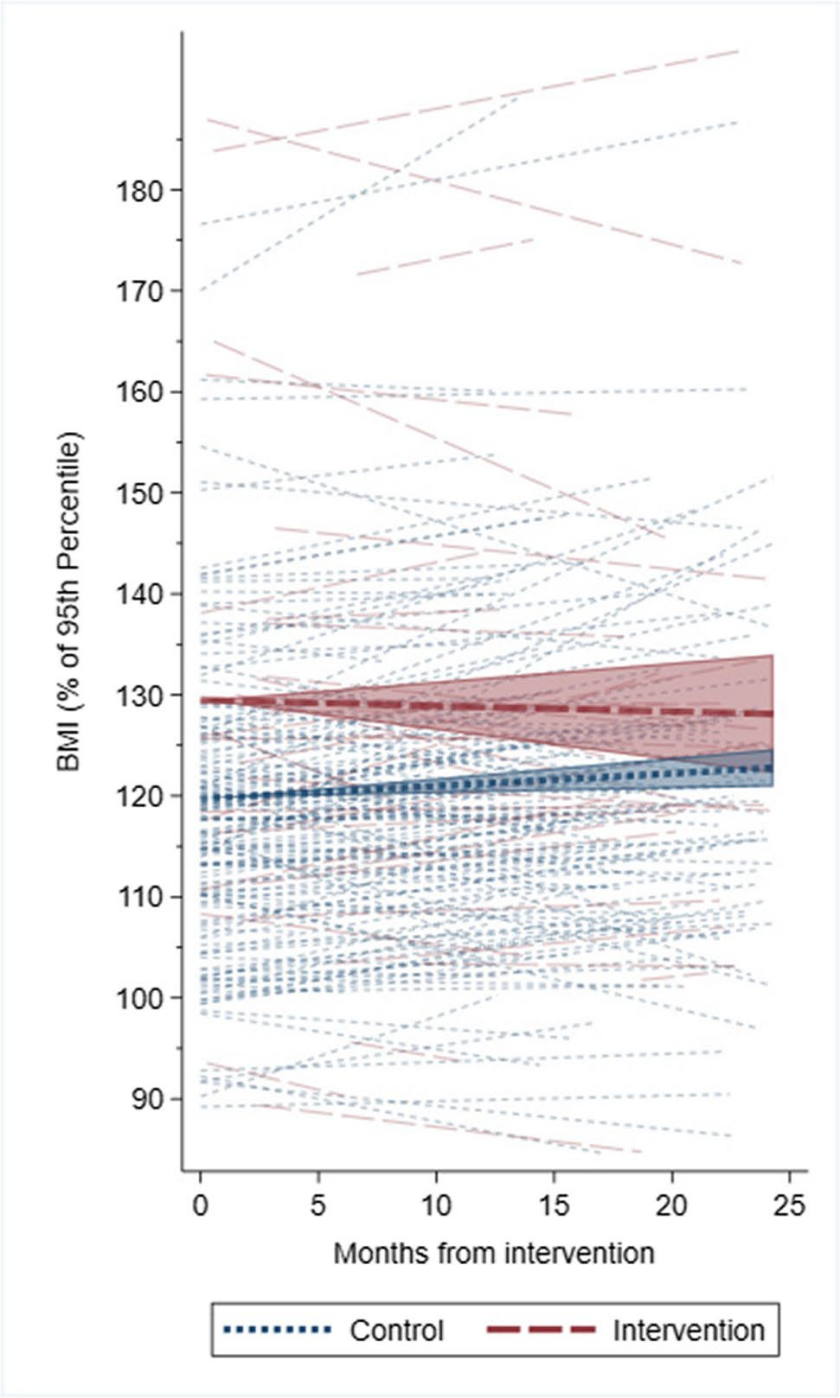
Spaghetti plots of predicted BMIpct95 values from the start of the intervention (time zero) to 24 months post-intervention for both the intervention and control participants. Predicted individual and average-by-intervention BMIpct95 slopes estimated with mixed effects linear regression model comparing BMIpct95 by intervention status, adjusting for patient demographics, baseline BMIpct95, allowing for patient-specific intercepts and patient-specific slopes, and allowing expected slopes to vary by intervention status. BMIpct95 is a measure normalized to the 95 th percentile of BMI. The predicted slopes of control participants are shown in blue/short dash, and intervention participants are shown in red/long dash. The bolded slope denotes the expected slope for the average participant within the control (blue, short dash) or intervention (red, long dash) groups, with banding showing the confidence interval. Intercepts for the average control and intervention participant were predicted using their intervention’s mean baseline BMIpct95

Overall, participation in the intervention group was associated with a lower BMIpct95 compared to controls (Coefficient − 1.01, 95% CI − 1.89, − 0.13) in a mixed effects linear regression model comparing BMIpct95 by intervention status, adjusting for participant demographics, baseline BMIpct95, allowing for participant-specific intercepts and participant-specific slopes, and allowing expected slopes to vary by intervention status (Fig. 2). Compared to controls, intervention participants had an additional decrease in BMIpct95 with each month post-baseline (Coefficient − 0.18, 95% CI − 0.35, − 0.02). When stratified by sex, participation in the intervention was associated with a lower expected BMIpct95 among females (Coefficient − 1.29, 95% CI − 2.49, − 0.09), but not males. We did not find a significant association with the intervention when we stratified by age group. When we stratified by age and sex, we found no significant difference in the expected change in BMIpct95 over time by intervention status.

#### Sensitivity Analysis (Full Results in Tables 3 and 4)

**Table 3:** Sensitivity Analyses Set One: Mixed multilevel regression model coefficients with their 95% confidence intervals comparing the BMIpct95 trend for the intervention and control participants adjusted for age at baseline, sex, and BMIpct95 at baseline

	Variable	*N*	Baseline BMI on or after intervention start			Drops observations after Feb. 28, 2020		
			Coefficient	95% confidence interval	*p*-value	Coefficient	95% confidence interval	*p*-value
Overall	Intervention	193	− 1.38	(− 2.42, − 0.35)	0.01	− 1.00	(− 1.99, − 0.01)	0.05*
	Months from intervention		0.13	(0.05, 0.20)	0.001	0.13	(0.05, 0.21)	0.002**
	Intervention * months from intervention		− 0.15	(− 0.32, 0.01)	0.07	− 0.23	(− 0.41, − 0.04)	0.02*
Females	Intervention	97	− 1.55	(− 2.94, − 0.16)	0.03	− 1.00	(− 2.25, 0.25)	0.12
	Months from intervention		0.11	(− 0.002, 0.22)	0.06	0.17	(0.05, 0.29)	0.01**
	Intervention * months from intervention		− 0.15	(− 0.39, 0.08)	0.20	− 0.28	(− 0.55, − 0.02)	0.04*
Males	Intervention	96	− 1.05	(− 2.57, 0.47)	0.18	− 0.72	(− 2.27, 0.84)	0.37
	Months from intervention		0.14	(0.04, 0.25)	0.01	0.09	(− 0.03, 0.20)	0.13
	Intervention * months from intervention		− 0.16	(− 0.40, 0.08)	0.20	− 0.21	(− 0.47, 0.05)	0.12
Age 4–6 years	Intervention	17	− 1.62	(− 4.23, 1.00)	0.23	− 0.87	(− 3.36, 1.62)	0.49
	Months from intervention		0.15	(− 0.06, 0.37)	0.16	0.15	(− 0.06, 0.37)	0.17
	Intervention * months from intervention		0.03	(− 0.45, 0.52)	0.89	− 0.04	(− 0.51, 0.44)	0.88
Age 7–10 years	Intervention	102	− 1.41	(− 2.83, 0.01)	0.05	− 1.08	(− 2.36, 0.19)	0.10
	Months from intervention		0.11	(0.01, 0.22)	0.04	0.11	(− 0.01, 0.23)	0.08
	Intervention * months from intervention		− 0.14	(− 0.37, 0.10)	0.26	− 0.18	(− 0.46, 0.10)	0.20
Age 11–14 years	Intervention	74	− 0.97	(− 2.71, 0.78)	0.28	− 0.55	(− 2.36, 1.26)	0.55
	Months from intervention		0.14	(0.02, 0.26)	0.03	0.15	(0.03, 0.27)	0.02*
	Intervention * months from intervention		− 0.22	(− 0.49, 0.06)	0.12	− 0.34	(− 0.63, − 0.05)	0.02*

^*^p-value ≤ 0.05, ** ≤ 0.01, *** ≤ 0.001

BMIpct95 is a measure normalized to the BMI 95 th percentile (BMI at the 95 th percentile would have a %BMIp95 of 100). In the first sensitivity analysis, we use observations on or after the intervention start date to calculate the baseline BMI (in the primary analyses, we used the observation closest to the intervention start date, whether before or after the intervention started). In the second sensitivity analysis, we dropped all BMIpct95 data after February 28, 2020, to minimize the effects of the COVID- 19 pandemic as a potential confounder

**Table 4:** Sensitivity Analyses Set Two: Mixed multilevel regression model coefficients with their 95% confidence intervals comparing the BMIpct95 trend for the intervention and control participants

	Variable	*N*	Includes only initial intervention inclusion criteria participants		
			Coefficient	95% confidence interval	*p*-value
Overall	Intervention	193	− 1.00	(− 1.94, − 0.05)	0.04*
	Months from intervention		0.11	(0.02, 0.20)	0.01**
	Intervention * months from intervention		− 0.15	(− 0.35, 0.04)	0.13
Females	Intervention	97	− 1.23	(− 2.50, 0.05)	0.06
	Months from intervention		0.09	(− 0.03, 0.20)	0.13
	Intervention * months from intervention		− 0.16	(− 0.41, 0.08)	0.19
Males	Intervention	96	− 0.71	(− 2.08, 0.67)	0.31
	Months from intervention		0.16	(0.02, 0.30)	0.03*
	Intervention * months from intervention		− 0.13	(− 0.45, 0.18)	0.40
Age 4–6 years	Intervention	17	− 1.53	(− 4.97, 1.92)	0.39
	Months from intervention		0.18	(− 0.05, 0.41)	0.12
	Intervention * months from intervention		− 0.03	(− 0.52, 0.46)	0.91
Age 7–10 years	Intervention	102	− 1.04	(− 2.26, 0.18)	0.09
	Months from intervention		0.11	(0.01, 0.22)	0.04*
	Intervention * months from intervention		− 0.17	(− 0.40, 0.06)	0.15
Age 11–14 years	Intervention	74	− 0.71	(− 2.24, 0.82)	0.37
	Months from intervention		0.06	(0.15, 0.28)	0.56
	Intervention * months from intervention		− 0.19	(− 0.68, 0.29)	0.44

^*^p-value ≤ 0.05, ** ≤ 0.01, *** ≤ 0.001

BMIpct95 is a measure normalized to the 95 th percentile of BMI (meaning that a patient with a BMI equal to the 95 th percentile would have a %BMIp95 of 100). The results of the third sensitivity analysis are shown. Only those who met the initial intervention inclusion criteria (age 5–12 years) were kept in the sample to examine the intervention effect within this group

First, in the model where we dropped observations after February 28, 2020, the interaction term between the expected BMIpct95 rate of change across the intervention group remained significant (Coefficient − 0.23, 95% CI − 0.41, − 0.04). Secondly, we restricted the baseline BMIpct95 measures to only those recorded on or after the intervention start date. In this case, the interaction term became insignificant but remained near the threshold for significance (Coefficient − 0.15, 95% CI − 0.32, − 0.01). Lastly, we only included participants who met the initial study inclusion criteria (*n* = 33) and found that while the trend of the association with the interaction term remained, it was no longer significant (Coefficient − 0.15, CI − 0.35, 0.04).

### Aim 2: Qualitative Evaluation to Identify Mechanisms and Barriers for Behavior Change

#### Interview Themes

Fourteen post-intervention interviews were completed with Spanish-speaking mothers. Four key themes emerged: (1) caregivers felt responsible for their children’s health; (2) families acquired new knowledge that they were able to directly apply through changes in household habits; (3) the group format facilitated mutual support and sharing among caregivers; and (4) the COVID- 19 pandemic exposed barriers to maintaining behavior change.

##### Theme 1: Caregivers Felt Responsible for Their Children’s Health (Table 5)

**Table 5:** Theme 1 quotes

*Caregivers felt responsible for their children’s health*
***Quote 1:**** Yo soy la que le estoy viendo por la comida de él, porque yo misma le compro en la tienda. Entonces yo tengo que estar pendiente en el peso de mi hijo. Es en la salud de mi hijo tengo que estar pendiente* I am the person who is choosing the food for my son because I myself buy it for him at the store. So I have to watch out for my son’s weight, for the sake of his health
***Quote 2:**** De principio era muy duro porque él no quería hacer ejercicio, él lloraba mucho, decía que no le gusta, que le duelen las piernas y que él no quería hacer. A veces lloraba y uno tenía que estar ahí con él, bueno, usualmente, yo para que mi hijo no se– a veces lloraba, entonces yo tuve que hacer el ejercicio. Yo hago ejercicio actualmente también con él, porque la Doctora Sarah me dijo que yo tenía que motivarlo a él* From the beginning it was very difficult because [my son] did not want to exercise, he cried a lot, and said he did not like it, that his legs hurt and that he did not want to do it. Sometimes he cried and one had to be there with him, well, usually, for my son I mean – sometimes he cried, so I had to exercise. I exercise at the moment also with him because Dr. Sarah told me I had to motivate him
***Quote 3****: Ella dice que ella bajó unos ahí en el internet, y dice que ella se pone a hacer ejercicio, porque no me deja que la mire, no le gusta que la mire uno, sí [risas]* [My daughter] says that she downloaded [exercises] here on the internet and says that she is starting to exercise, because she did not let me watch her, she does not like anyone watching her, yes [laughs]

Following the intervention, many parents described themselves as gatekeepers for their children’s nutrition, as they bought and prepared meals for the household. Moreover, they took on the role of motivating their child to exercise, which often meant leading their family in physical activity. Overseeing their child’s healthy habits was more challenging for parents of older youth due to their growing independence.

From the beginning it was very difficult because [my son] did not want to exercise, he cried a lot, and said he did not like it, his legs hurt, and he did not want to do it... so I exercised with him because [the AHF physician instructor] told me I had to motivate him [Translated from Spanish].

##### Theme 2: The AHF Program Provided Families with New Knowledge That They Applied to Change Household Habits (Table 6)

**Table 6:** Theme 2 quotes

Families acquired new knowledge that they applied to change household habits
*Antes yo no comía muchas semillas secas, frutas secas, porque tal vez no me gustaban o no me llamaba la atención tenerlas, pero ahora sí, compramos. A veces comprábamos otras cosas para tener, como snacks, pero ahora compramos más frutas secas que otros, como por ejemplo, los chips o cosas dulces. También el cambio de sodas, tomar más agua y no sodas* Before, I did not eat many seeds [or] dried fruit maybe because I did not like them or because it was not brought to my attention to have them, but yes, we buy them now. Sometimes we bought other things to have like snacks, but now we buy more dried fruit than other things, like for example, chips or sweets. Also the change with sodas: we drink more water and no soda
*Sí, hemos hecho, gracias a Dios, muchos cambios. Lo que hablamos es: “Esto ya no lo vamos a traer. Las sodas ya no van a estar en la casa porque es daño”, y ya se entiende lo que no se va a traer. Por ejemplo, quitamos los chips, que se traían muchos a la casa, ya tampoco vienen a la casa. Ellos han entendido bastante* We have made, thank God, many changes. What we say is: “We are no longer going to get this. The sodas are no longer going to be in the house because they are harmful.” And it is understood that [these things] will not be brought in. For example, we got rid of the chips, which were brought in often to the house, and now they do not come to the house either. [The kids] have understood quite a bit
Antes nosotros no consumíamos tantos vegetales, y ahora yo, desde que comencé con el programa, yo me puse la meta de incluir vegetales en cada comida. Y ahora estamos así, ya no solo incluyo uno, incluyo dos vegetales combinados en el plato de la comida de todos, de la familia, porque antes nada más eran los niños y yo, y poco a poco se fue integrando mi esposo, porque era el que un poquito más [risas]– como quien dice [diafonía]. Entonces, ya ahora al vernos, ya también, y el más pequeñito también, no le gustaba comer muchos vegetales, y ahora ya también come Before, we were not consuming so many vegetables, but now since I began with the program, I set a goal to include more vegetables in each meal. And now we eat like this, including not only one,[but two vegetables combined in each plate of food for everyone in the family. Before it was just the children and me and little by little my husband was integrated […] and the littlest one also. He did not like to eat many vegetables, but now he also eats them
*A veces hacemos cuatro días a la semana o hay veces que hacemos, a veces, hasta cinco* *días a la semana. Lo llevo al parque y damos una caminada, corremos con una pelota o* *cosas así. Y las otras veces aquí en la casa, hacemos como zumba o hacemos el yoga kids, un programa muy bueno. Tenemos también una bicicleta de piso, y también ellos* *están en bicicleta. Yo los apoyo y hacemos [inaudible] y cosas así* Sometimes we do four days a week or there are times that we do, sometimes, up to five days a week. I bring [my son] to the park and we take a walk, we run with a ball or things like that. And the other times here in the house, we do Zumba or YogaKids, a very good program. We also have a stationary bike, and they also get on the bike. I support them and things like that

Key knowledge parents retained from the program included learning to read nutrition labels with a new awareness of the sugar content of certain beverages and cereals as well an appreciation of appropriate portion sizes. Many applied their new knowledge to make healthier substitutions in their diet and drinking behaviors and incorporated greater physical activity into their routine. They linked these behavior changes to knowledge gained in the AHF program. Although not all knowledge and behavior changes were maintained, parents commonly reported sustained habit changes for both the child and family that became the new normal and positively impacted their family’s health.

We have made, thank God, many changes. What we say is, ‘We are no longer going to get this. The sodas are no longer going to be in the house because they are harmful’ we got rid of the chips, which were brought in often to the house... [The kids] have understood quite a bit [Translated from Spanish].

##### Theme 3: The Group Format Facilitated Solidarity and Mutual Support Among Caregivers Through the Sharing of Struggles and Common Experiences (Table 7)

**Table 7:** Theme 3 quotes

The group format facilitated solidarity and mutual support among caregivers through the sharing of struggles and common experiences
***Quote 1:**** Compartíamos los padres de familia, que a veces eso nos ayuda a cómo uno tiene que actuar con los niños. A veces, muchos padres se sentían frustrados, que los niños subían de peso y que caían en depresión, solo querían comer. Diferentes padres de familia, teníamos diferentes testimonios, como era el trabajo con los niños cuando están en ese problema* The parents of the families shared [their experiences with each other], which at times helps us with how to work with the children. Sometimes, many parents felt frustrated, that the kids were gaining weight and falling into depression, that they only wanted to eat. [As] parents of different families, we had different testimonies, working with the kids when they are dealing with that issue
***Quote 2:**** Creo que también lo que me gustó es que conoces más personas y ves muchas opiniones diferentes. Y no sé, como que vas acostumbrándote a las personas, a ver cómo tienen diferentes pensamientos. Los niños también, a veces son chistosos en hablar, dicen cosas. Yo creo que también es bonito conocer. Se conoce la gente, como que conoces diferentes familias, modos de vida. Y las personas que hicieron el programa son buenas, te ayudan. No es un grupo como que para juzgar, solo es como para ayudarnos. Me gusta. Me gustó mucho conocer más gente también* I think that also what I liked was that you get to meet more people and see many different opinions. You become familiar with these people [and] see how they have different thoughts. The children were also funny sometimes when they were talking, saying things. I believe that it is nice to meet [others]. One gets to know the people, like you get to know different families [and] ways of living. And the people who made the program are great. They help you. It is not a group for judging but for helping us. I like it. I liked getting to know more people too

Since there was a consistent group over multiple sessions during the AHF intervention, parents were able to get to know each other and create a sense of community. They described an atmosphere of solidarity and mutual growth where they shared and learned from each other to overcome challenges in establishing healthier family habits.

The parents of the families shared with each other, which helps us to know how to work with the children. Sometimes, many parents felt frustrated, that the kids were gaining weight and falling into depression, that they only wanted to eat. [As] parents of different families, we had different testimonies, working with the kids when they are dealing with that issue [Translated from Spanish].

##### Theme 4: The COVID- 19 Pandemic Exposed Barriers to Maintaining Behavior Change (Table 8)

**Table 8:** Theme 4 quotes

The COVID- 19 pandemic exposed barriers to maintaining behavior change
*Al principio fue un poco difícil, influenció mucho porque ellos, si están más en casa, ellos comen* *más. Anteriormente yo hacía la compra cada 15 días, y llegó un momento en cinco meses, que yo tenía que comprar cada semana porque la comida estaba como faltando en la casa, porque se comía mucho la comida muy rápido. Pero después, conforme iban pasando los meses, otra vez volvimos a la normalidad. Como que fue un tiempo, como ansiedad, fue una ansiedad que ellos tenían por tener algo en la boca. Por todo lo que estaba pasando, porque no salíamos, porque solo pasábamos en casa y estudiando desde la computadora. Entonces, nosotros pasamos diciéndoles lo importante que es que ellos no coman tanto dulce, que mejor coman frutas* At first it was a little difficult, [the pandemic] influenced a lot because [the kids], if they were in the house more, they ate more. Before, I went grocery shopping every 15 days, and there was a moment 5 months [into the pandemic] that I was having to shop every week because food was lacking in the house because it was being eaten very quickly. But after that, as the months were going by, we returned again to normality. Like there was a time, [when there was] anxiety that [the kids] needed to have something in their mouth. It was happening to everyone because we were not going out, because we were only staying in the house and going to school via the computer. So we told them that the important thing is that they do not eat too many sweets, that it is better to eat fruits
*El ejercicio ya es difícil porque no quieren salir, menos ahorita con la pandemia. Cuando estábamos en el programa hacíamos bastante ejercicio, pero ahorita con lo de la pandemia como que nadie quiere salir ni a caminar a la cuadra, y como ahorita es más difícil por el horario, porque como que se oscurece más temprano, entonces ya no salimos* Exercising is difficult because [the kids] do not want to go out, and even less now with the pandemic. When we were in the program, we did quite a bit of exercise, but now with the pandemic no one wants to go out or walk around the block, and now it is more difficult because of the [winter], because it gets dark earlier, so we do not go out

Parents noted that the COVID- 19 pandemic impacted diet and physical activity as their children snacked more and moved less, contributing to weight gain. Some participants described how they tried to incorporate the knowledge they gained during the AHF intervention to stabilize their child’s weight and adjust to a new normal. Online resources for maintaining physical activity (e.g., YouTube yoga videos) were helpful during quarantine, but families preferred outdoor exercise that was limited by social distancing.

[The COVID- 19 pandemic] influenced a lot, if [the kids] were in the house more, they ate more. Before, I went grocery shopping every 15 days, and there was a moment 5 months [into the pandemic] that I was having to shop every week.... as the months were going by, we returned to normality… we told them the important thing is that they do not eat too many sweets, that it is better to eat fruit [translated from Spanish].

## Discussion

In this mixed methods study, the Baltimore AHF intervention participants had a greater reduction in their BMI percentile compared to those in the control group at two years post-intervention. The intervention was well-received, and many parents voiced that they used new knowledge and social support from the program to implement household behavior changes related to diet and physical activity for themselves and their child. Our findings build on the existing evidence base for the original AHF intervention. First, our results suggested that AHF participation may have long-lasting effects two years post-intervention. Second, it was feasible and efficacious to apply AHF in alternative settings with emerging Latinx communities where there may be less infrastructure and social supports for Spanish-speaking families.

These findings align and build on the existing body of literature on pediatric obesity prevention and treatment interventions for Hispanic/Latinx youth. A recent meta-analysis of 67 randomized and nonrandomized controlled pediatric obesity trials for Hispanic/Latinx youth estimated an overall intervention effect of − 0.15 (95% CI − 0.20, − 0.10) based on the standardized mean difference for the BMI between the intervention and control groups, indicating a modest yet statistically significant reduction in BMI among participants in intervention groups compared to controls [5]. However, this meta-analysis was constrained by the absence of long-term post-intervention data, relying solely on BMI measurements taken immediately after the intervention [5]. Our results similarly demonstrate a modest reduction in BMI among the intervention group but also address an important literature gap by contributing to the limited body of research examining the long-term impacts of such programs on BMI trends among Hispanic/Latinx youth. Moreover, existing intensive community-based programs that meet USPSTF recommendations to address childhood obesity, such as the MEND (mind exercise nutrition do it!) program, have shown limited effectiveness in reducing BMI among primary Spanish speakers compared to primary English speakers [20]. These programs also experience higher dropout rates among families with non-English home languages and lower levels of parental education—factors that are prevalent among immigrant Latinx parents [20]. In contrast, our study findings suggest that the AHF program was both feasible and acceptable and associated with BMI reductions among primary Spanish-speaking parents within immigrant families. Our findings offer additional evidence for a culturally responsive pediatric obesity intervention with possible long-term effects to help ensure that Latinx children are not overlooked in the application of USPSTF recommendations for addressing childhood obesity.

Our post-intervention interviews offered insights into potential mechanisms for the intervention’s lasting effectiveness in an emerging Latinx community. Parents voiced that intervention participation increased their knowledge and parent efficacy as a role model and leader within their household, creating an opportunity for social support and information exchange with other parents. The family-based nature of the program reinforced the parents’ sense of responsibility to share how to lead a healthy life with their child and encourage behavior change. Notably, fathers rarely attended AHF sessions. It may be worth exploring in future studies how to integrate more family members into the program [21]. Prior studies found that fathers face additional barriers to participation including competing work commitments, reduced program awareness, social discomfort with a mother-dominated group, perceived passive role in their child’s weight management, and not being concerned about their child’s weight [22]. Future work could also explore how household and caregiver composition (e.g., single parent) influences program impact. Collectively, these findings suggest that effective pediatric obesity intervention programs for emerging Latinx communities may benefit from a culturally sensitive, family-centered intervention that promotes knowledge transfer within small groups [23, 24].

While AHF shows promise, our findings also suggest a need for caution in relying solely on the AHF program to meaningfully impact pediatric obesity in this population. The magnitude of the effect on BMI was small. This supports the large body of evidence that multi-level interventions are needed to address structural factors contributing to barriers to healthy lifestyle choices [25, 26]. Regardless, our findings suggest that AHF not only allowed the children to maintain their weight but also showed a reduction in their weight compared to usual care. This is meaningful and can be considered one component in a broader effort to reduce pediatric obesity. For example, a randomized control trial found that a community-based, multi-level, multi-setting, multi-component intervention model reduced BMI trajectory for Latinx children with overweight and obesity over 1 and 2 years compared to a nutrition and health education control intervention alone [27]. AHF could be embedded within such a multi-level intervention. Expanding access to such programs can help reduce barriers facing Latinx immigrant families in finding comprehensive, culturally and linguistically tailored obesity management options.

Our study also offers an opportunity to understand barriers to maintaining behavior change following a lifestyle intervention through the lens of the COVID- 19 pandemic. The association between the intervention and BMI was stronger after removing BMI observations following February 28, 2020. Caregivers also voiced difficulty maintaining motivation to sustain behavior change, particularly during the COVID- 19 pandemic [28]. This suggests a need for additional touch points or booster sessions, especially during times of stress and alterations in a family’s typical routine. Per caregivers, further capacity building may be needed to address snacking, how to remain active while indoors, and support older youth in behavior change.

These findings should be interpreted in light of the study’s limitations. First, selection bias is possible—more motivated families with access to resources may be more likely to engage in the intervention compared to the families in the matched control group. The baseline BMIpct95 was higher in the intervention compared to the control group despite attempts at matching, suggesting that the intervention was potentially more attractive to clinicians and/or families with children with the highest BMIs. Similarly, post-intervention interviews occurred only with a subset of parents; parents who were least likely to benefit from the intervention may not have been interested in being interviewed. Second, as a pragmatic trial, there was no consistent record of the children who were referred but did not attend any of the intervention sessions or the number of sessions each child completed. Third, individual BMIpct95 measures were not collected on the same days in relation to the intervention start date. Reassuringly, the coefficients were similar when alternative definitions were used to identify the baseline BMIpct95. Fourth, it is possible that the degree of follow-up appointments differed between the intervention and control groups, although that was not readily evident in our analyses.

Future research would benefit from using a randomized control trial design with a larger sample for subgroup analyses while maintaining longitudinal follow-up. For instance, it is important to determine whether the sex differences in the BMIpct95 trends are replicated in large sample sizes and if so, why the intervention may be more impactful for girls. Moreover, it may be helpful to track whether there are differences in the types of children who choose to participate and drop out of the program as well as program implementation strategies, as implementation may vary across communities and vary in effectiveness.

There are key implications for pediatricians, pediatric obesity researchers, and policy makers. AHF or similar family-centered interventions that offer health education, and create opportunities for social support may be an option to meet the AAP clinical guideline recommendation for comprehensive, intensive health behavior and lifestyle treatment for Latinx families. To improve its effectiveness, the AHF intervention may need adaptation and further testing of strategies to engage more fathers for male role modeling, develop booster capacity building sessions to help families sustain healthy lifestyle changes as challenges arise, and modifications to meet the needs of older children and adolescents. AHF should be one component of a multi-prong approach as it is likely not sufficient to move the needle on the rates of pediatric obesity for the Latinx community.

## Conclusion

This mixed-method study evaluates the long-term outcomes of an evidence-based intervention for Latinx families and incorporates parent interviews to ensure findings are grounded in participant experiences. The results suggest the need for ongoing support of families in the AHF program over time and combining the program with other pediatric obesity efforts to ensure effective and sustainable solutions to improve the weight status of Latinx children in emerging communities. Development and dissemination of effective programs for this population may help to reverse their disproportionately high rates of childhood overweight/obesity and its associated complications.

## Data Availability

The data that support the findings of this study are not openly available due to reasons of sensitivity and are available from the corresponding author upon reasonable request and institutional review board approval. Data are located in controlled access data storage at Johns Hopkins.
