# Correction: Two-Year Outcomes for the Active and Healthy Families Pediatric Obesity Group Intervention for Families in an Emerging Latinx Community: a Mixed Methods Study

**DOI:** 10.1007/s40615-026-02894-3

**Published:** 2026-02-24

**Authors:** Jaime La Charite, Lisa Ross DeCamp, Laura Prichett, Amanda Grace Finney, Jenny Y. Chen, Albert E. Holler, Yoon Ji Moon, Alexa Mullins, Rafael Ospino, Kori Porosnicu Rodriguez, Sarah Polk

**Affiliations:** 1 The Johns Hopkins University School of Medicine, 601 N Caroline St, Baltimore, MD USA; 2 Department of General Internal Medicine, University of California, Los Angeles, 1100 Glendon Ave. Suite 900, Los Angeles, CA 90024 USA; 3 Children’s Hospital Colorado, Aurora, CO USA; 4 Department of Pediatrics, University of Colorado School of Medicine, Aurora, CO USA; 5 Adult and Child Center for Outcomes Research and Delivery Science, Aurora, CO USA; 6 Department of Pediatrics, Johns Hopkins University School of Medicine, Baltimore, MD USA; 7 Department of General Pediatrics, Children’s Hospital of Philadelphia, 3401 Civic Center Boulevard, Philadelphia, PA 19104 USA; 8 Department of Pediatrics, Children’s Hospital of Philadelphia, Philadelphia, PA USA; 9 Department of Neurology, Johns Hopkins University School of Medicine, 600 North Wolfe Street, Baltimore, MD USA; 10 Centro SOL, Johns Hopkins University, 5200 Eastern Ave, Baltimore, MD 21224 USA; 11 Center for Salud, Health and Opportunity for Latine, Baltimore, USA

**Correction: Journal of Racial and Ethnic Health Disparities**

10.1007/s40615-025-02420-x

The surname of coauthor Kori Porosnicu Rodriguez was not fully presented in the article as originally published.

The original article has been corrected.

